# ^13^C Labeling of Nematode Worms to Improve Metabolome Coverage by Heteronuclear Nuclear Magnetic Resonance Experiments

**DOI:** 10.3389/fmolb.2019.00027

**Published:** 2019-04-26

**Authors:** Florian M. Geier, Armand M. Leroi, Jacob G. Bundy

**Affiliations:** ^1^Department of Surgery and Cancer, Imperial College London, London, United Kingdom; ^2^Department of Life Sciences, Imperial College London, South Kensington, London, United Kingdom

**Keywords:** *C. elegans*, HSQC, CT-HSQC, sensitivity, metabolomics, metabolome, NMR metabolomics, labeling

## Abstract

Nuclear magnetic resonance (NMR) spectroscopy is widely used as a metabolomics tool, and 1D spectroscopy is overwhelmingly the commonest approach. The use of 2D spectroscopy could offer significant advantages in terms of increased spectral dispersion of peaks, but has a number of disadvantages—in particular, heteronuclear 2D spectroscopy is often much less sensitive than 1D NMR. One factor contributing to this low sensitivity in ^13^C/^1^H heteronuclear NMR is the low natural abundance of the ^13^C stable isotope; as a consequence, where it is possible to label biological material with ^13^C, there is a potential enhancement of sensitivity of up to around 90fold. However, there are some problems that can reduce the advantages otherwise gained—in particular, the fine structure arising from ^13^C/^13^C coupling, which is essentially non-existent at natural abundance, can reduce the possible sensitivity gain and increase the chances of peak overlap. Here, we examined the use of two different heteronuclear single quantum coherence (HSQC) pulse sequences for the analysis of fully ^13^C-labeled tissue extracts from *Caenorhabditis elegans* nematodes. The constant time ct-HSQC had improved peak shape, and consequent better peak detection of metabolites from a labeled extract; matching this against reference spectra from the HMDB gave a match to about 300 records (although fewer actual metabolites, as some of these represent false positive matches). This approach gives a rapid and automated initial metabolome assignment, forming an ideal basis for further manual curation.

## Introduction

NMR metabolomics has been widely applied to the model organism *Caenorhabditis elegans* for the elucidation of all kinds of biological problems, including phenotyping of gene function (Atherton et al., 2008; Fuchs et al., 2010; Pontoizeau et al., 2014), response to toxicants (Hughes et al., 2009; Zeitoun-Ghandour et al., 2011; Jones et al., 2012), analysis of secondary metabolites and receptor ligands (Pungaliya et al., 2009; Izrayelit et al., 2013; Mahanti et al., 2014), and analyzing basic biological processes such as diet and growth (Swire et al., 2009; Reinke et al., 2010). NMR has generally lower sensitivity compared to mass spectrometry-based metabolomics methods, and so 1D NMR analysis typically gives information on only a few tens of metabolites. NMR metabolomics does have a number of intrinsic advantages (Markley et al., 2017), though. Pre-eminently, the genuinely untargeted nature of NMR detection means that it is a near-universal detector for all kinds of small molecules from all kinds of chemical classes. In addition, though, the advantages include the rich spectral information that is often available for unknown or novel metabolites; extremely high instrument precision, that allows detection of even small changes in metabolite abundance; and the ability to extend the information available from NMR by extending the 1D spectra most often used for profiling to multidimensional spectra, or by otherwise modulating the NMR response (for instance, by editing the spectral intensity as a function of molecular weight, to enable better analysis of both metabolites and lipoproteins in blood plasma samples). The usefulness of 1D NMR is limited not only by absolute metabolite concentrations, but also, often, by resonance overlap (Tredwell et al., 2011; Sokolenko et al., 2014). Two-dimensional NMR experiments can greatly increase peak dispersion, and hence the number of potentially resolvable resonances. Heteronuclear single quantum coherence (HSQC) experiments (Bodenhausen and Ruben, 1980) are not only key tools for structural assignments and elucidation, but are also highly useful for analysis of metabolite mixtures owing to the wide chemical shift range of the ^13^C axis (Xi et al., 2008). However, its major drawback is its low inherent sensitivity (compared to the 1D experiments frequently used for profiling), and so it mostly yields information on high abundance metabolites; which often are well known already. Biologically interesting, lower-concentration peaks remain difficult to assign. High-biomass samples (e.g., with >50 mg extract dry weight) can be analyzed in relatively short times simply by making very concentrated samples (Lewis et al., 2007), but this is often difficult to accomplish for *C. elegans* samples.

The low sensitivity of the HSQC experiment can be mitigated by incorporating ^13^C into the metabolome, as ^13^C has a low natural abundance of only 1.1%. Kikuchi et al. (2004) were the first to ^13^C and ^15^N label *Arabidopsis thaliana* to probe ethanol stress response and nitrogen fluxes during germination, respectively; both with HSQC pulse sequences. Chikayama et al. (2008) built an online database of >1000 ^13^C–^1^H HSQC chemical shifts they had assigned to 142 metabolites from *Arabidopsis thaliana* and silkmoth larvae (*Bombyx mori*), and also implemented an algorithm that automatically considers multiple HSQC resonances and assigns plausible metabolites based on a statistical likelihood value (Chikayama et al., 2010). This approach has also been applied to *C. elegans*: An et al. (2012) used HSQC profiling with ^13^C enriched *C. elegans* samples to identify metabolic changes in a *sir-2.1* mutant, although they used a 3D NMR approach with an additional TOCSY sequence. Indeed, ^13^C labeling of *C. elegans* has even been used to acquire INADEQUATE spectra of metabolites (Clendinen et al., 2015a). There are now an increasing number of studies that have used HSQC experiments, and a number of variations thereof, for analysis of metabolites in biological samples, including both natural-abundance and labeled or partially labeled biomass samples (Soong et al., 2014; Clendinen et al., 2015b; Mobarhan et al., 2016; Nath et al., 2016; Schätzlein et al., 2018).

With full ^13^C abundance, carbon – carbon couplings, which at natural abundance are rare events, become an issue. Classic broadband decoupling techniques used to suppress ^1^H–^13^C satellites do not work for ^13^C–^13^C coupling at full ^13^C incorporation (Vuister and Bax, 1992), and would lead to unacceptable sample heating. To this end, the structural NMR community has developed pulse sequences that circumvent C-C couplings. In particular, the constant time HSQC (ct-HSQC), proposed by Vuister and Bax (Vuister and Bax, 1992), is often used for fully labeled multidimensional protein NMR spectroscopy (Mandal and Majumdar, 2004). A ct-HSQC pulse sequence is very similar to a standard HSQC sequence. However, instead of the usual variable ^13^C evolution period t_1_, a constant time is used. Also during this interval, an additional 180° pulse refocuses ^13^C–^13^C coupling constants. Therefore, couplings will not split but appear as cross-peaks along the F_1_ domain and (ideally) not interfere with the rest of the spectrum.

Therefore, this experiment aims to evaluate whether ^13^C labeling and the use of a ct-HSQC pulse sequence is a valuable approach to improve annotation of *C. elegans* HSQC visible metabolites.

## Materials and Methods

### Sample Generation

An unlabelled control sample was prepared by growing wild-type N2 worms on agar plates, according to standard procedures. To generate the ^13^C enriched *C. elegans* samples, agar plates were prepared without peptone, using the pellet of a 25 ml overnight culture of the *E. coli* K-12 wild type strain NCM3722 spread onto the agar plates. NCM3722 was grown in a minimal medium, with ^13^C-glucose and ^14^N-ammonium chloride as sole carbon and nitrogen source, respectively. To allow full isotope enrichment, worms were grown over two generations on the isotope medium.

All worm cultures (isotope enriched and unlabeled) were age synchronized. Worms were harvested as young adults and extracted by bead-beating in 80% methanol, according to a consensus protocol based on a comparison of different metabolite extraction methods (Geier et al., 2011). The extracts were centrifuged (16,000 g, 10 min) and the supernatants dried under reduced pressure, and then stored at −80° C until analysis.

In both cases, the biomass of three plates of worms were pooled to yield one biological replicate, in order to improve the signal to noise ratio.

### Acquisition and Processing

For NMR spectroscopy, the samples were prepared by rehydrating each dried extract in an aqueous (D_2_O) phosphate buffer (0.1 M, pH 7). Acquisition was performed at 300 K on a Bruker Avance II spectrometer at 800 MHz ^1^H observation frequency. A cryogenically cooled 5 mm triple-axis inverse probe was used. The system was controlled by Bruker TopSpin 2.1. For the regular HSQC spectra a Bruker gradient HSQC sequence with pre-saturation and sensitivity enhancement was used. The constant time sequence (ct-HSQC) used the Bruker ”hsqcctetgpsp” sequence, adapted from Isaacson et al. (Isaacson et al., 2007) and originally based on Vuister and Bax (Vuister and Bax, 1992). The constant t_1_ evolution time needs to be optimized, to target the coupling constant that needs to be “removed” during the 180° refocusing pulse. As most metabolites of a complex mixture have at least some resonances in the aliphatic region, it was decided to target those. Aliphatic C-C couplings are usually around 32-40 Hz. Therefore, the constant time (d_23_) was set to (2T = 1/*J* = 1/38.5 Hz = 26.6 ms). The pulses in the carbon domain were adiabatic, shaped pulses. For both the HSQC and ct-HSQC the 90° pulse p_1_ was set to 14 ms. After 32 dummy scans, 64 time domain transients with 1024 points in the F_2_ domain and 512 points in the F_1_ domain were acquired. The spectral sweep width was 10 ppm in the proton and 90 ppm in the carbon domain, centered at 4.70 and 90 ppm, respectively. The acquisition time was 102 ms, and a GARP4 decoupling sequence was used.

For processing inside TopSpin 3.1, the FID was multiplied by a Gaussian window function in the F2 and a shifted sine-bell squared function in the F1 dimension. The time domain spectrum was then Fourier transformed into a 1 k by 1 k data point frequency spectrum. Phase correction was performed by optimizing the phase angle of 1D projections of selected peaks across the whole spectrum.

### Spectral Deconvolution and Automatic Database Matching

For automatic assignment, the processed spectra were loaded into rNMR (Lewis et al., 2009). The signal/noise threshold was manually adjusted so that peaks had defined contours and no random noise was visible in spectral regions that are unlikely to contain peaks, i.e., the high proton, low carbon range. Peaks were detected using the default algorithm, without prior signal smoothing. In the constant time HSQC spectra, peaks stemming from the F_1_ artifact were manually removed. For assignment, the HSQC reference spectra from HMDB (Wishart et al., 2007, 2009, 2012, 2017) were used. (Worm-specific databases Witting et al., 2018 were not available when the work was carried out.) To this end all available spectra (*n* = 669) were downloaded (on the 21.11.2012) as XML files and read into R. Spectra were filtered by meta-information and only spectra recorded in similar experimental conditions were kept (aqueous buffer with pH ≈ 7).

As the reference spectra were recorded in a similar but not identical setup, ppm values can slightly deviate. This deviation might be composed of a systemic and an individual peak-by-peak component. In this experiment it was attempted to minimize both. The systemic shift was estimated by comparing ppm shifts of 15 peaks from different metabolites across the spectrum with the ppm values of the corresponding reference peaks.

Peak to peak deviations are more difficult to deal with. When matching the spectra against a reference, the acceptable error must be large enough to allow for the peak to peak deviation. However, when the allowed error margin is too large, one peak may match other neighboring peaks, making the match ambiguous and therefore reducing the power of the query. Therefore, it is important to adapt the error tolerance for each dataset, when an automatic search algorithm is used (Chikayama et al., 2010). The best way to find this value would be to annotate most metabolites in the mixture and determine their (median) distance the reference. However, this tedious approach is contradictory to the intention behind automatic assignment, which the goal of this experiment. A Monte-Carlo simulation was used to optimize these parameters for both proton and carbon shifts.

The selected cross peaks and the HMDB HSQC reference peaks were matched three thousand times. Each time, the allowed ppm error was randomly chosen within a window of 1 to 0.001 ppm and 2 to 0.005 ppm in the proton and carbon domain, respectively. After each iteration, it was counted (a) which fraction of metabolites from the HMDB reference spectra did not receive at least one match from a HSQC spectral peak. (b) how many HMDB metabolites were completely matched (each cross peak of the reference matched a cross peak of the spectrum). The convolution of (a) and (b), (a * b), was then calculated to determine the best trade off between the minimum (a) and maximum (b) matching window size, necessary to minimize false negatives and false positives, respectively. The maximum (best tradeoff) of the spectrum that resulted from the convolution determined the error margin for the final matching of HSQC peaks and HMDB reference spectra.

## Results and Discussion

### ^13^C Enrichment can Achieve a Drastic Increase in Sensitivity

The first step in evaluating whether ^13^C enriched samples acquired by ct-HSQC pulse sequences for *C. elegans* metabolite assignment are valuable, is to compare a fully ^13^C labeled vs an unlabelled sample. To this end, wild type worms were either fed with *E. coli* grown on U-^13^C-glucose or unlabelled glucose minimal medium. D_2_O reconstituted 80 % MeOH extracts of three pooled plates were then compared using ct-HSQC sequences. To make the spectra comparable, the signal-to-noise cut-off of both spectra was scaled such that the ^1^H resonances at 5.20 ppm (from the anomeric protons of trehalose) matched each other's intensity.

As expected and documented in the literature (Kikuchi et al., 2004), an increase in signal intensity obtained from isotope enrichment of the matrix is immediately obvious (Figure S1). Not only do cross peaks that are already visible in the ^12^C extract become more intense, many new resonances appear above the detection limit, especially in the otherwise sparse aromatic region; the aliphatic region also increases in resonance density. Focussing in on a smaller chemical shift region shows that the peaks are still generally well resolved, with only a few instances of peak overlap even in the most crowded region of the spectrum.

### The Constant Time Version has Some Advantages Compared to the Regular HSQC Pulse Sequence for ^13^C Enriched Samples.

**Figure 1:**
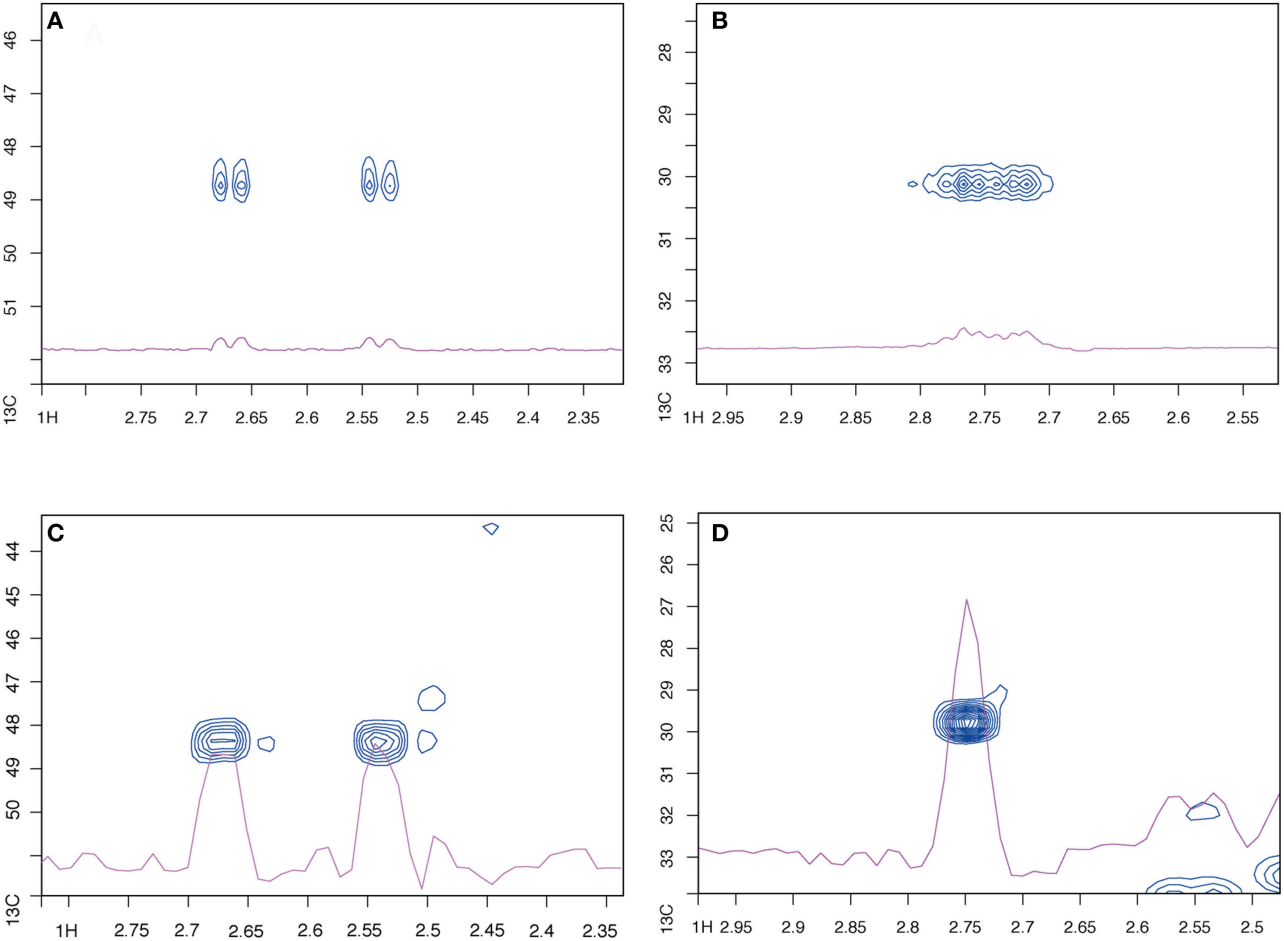
The constant-time HSQC has better peak shape for fully ^13^C labeled *C. elegans* extracts than the standard sequence. **(A,B)** Show two selected peaks using a standard HSQC, **(C,D)** show the same peaks, respectively, with the ct-HSQC.

If the spectra from the constant time and regular HSQC sequences are compared, they initially look rather similar, especially at full resolution (Figure S1), except that the ct-HSQC spectrum contains additional artifacts. These appear at the central ^13^C frequency, so may well be caused by incorrect calibration of the ^13^C pulses (the ^13^C pulse power levels were not optimized for this specific sample, in this case). Luckily these only affect a region of the spectrum containing little or no information. At a higher magnification, though, the benefits of the constant time sequence become apparent. When examining the aliphatic region alone, the ct-HSQC peaks have an improved peak shape (Figure S2). Examining individual peaks makes this even clearer. There are many examples where carbon splitting patterns collapse into a single cross peak. Two illustrative examples are shown in Figure 1. By removing C-C coupling, two doublets and one high-order multiplet are collapsed into two and one singlets, respectively. This peak simplification will help automated assignment approaches, and will also increase the sensitivity of the experiment by avoiding peak splitting. NB that the increased resolution here is in the F2 (^1^H) dimension: this is surprising, but perhaps caused by the constant time filter acting as a partial relaxation filter, leading to increased resolution.

There are also some potential disadvantages of the ct-HSQC sequence: one point is that the signal intensity is *J*-modulated. However, this should primarily affect comparison between different nuclei (either between or within metabolites). It should not be a problem when studies are essentially qualitative, as for our current study (except in the situation where peaks from particular nuclei might fall below detection limits), and even for quantitative studies, it should not affect comparison between spectra. A second point is that the absolute sensitivity is reduced: for example, focussing on one specific spectral region, and comparing the standard and ct-HSQC, there is an additional resonance visible (at 6.06/88.9 ppm) in the standard spectrum (Figure S3). Clearly, low-intensity metabolite signals may be lost.

A number of software approaches for automated metabolite assignment from 2D spectra exist, both for HSQC experiments alone (Lewis et al., 2007; Bingol et al., 2015) and also for other 2D spectra (Robinette et al., 2008; Bingol et al., 2012). Nonetheless, assignments are still often performed manually. Fully automated assignment would be highly desirable, considering both the amount of work involved in manual annotation, and the potential for human error.

**Figure 2:**
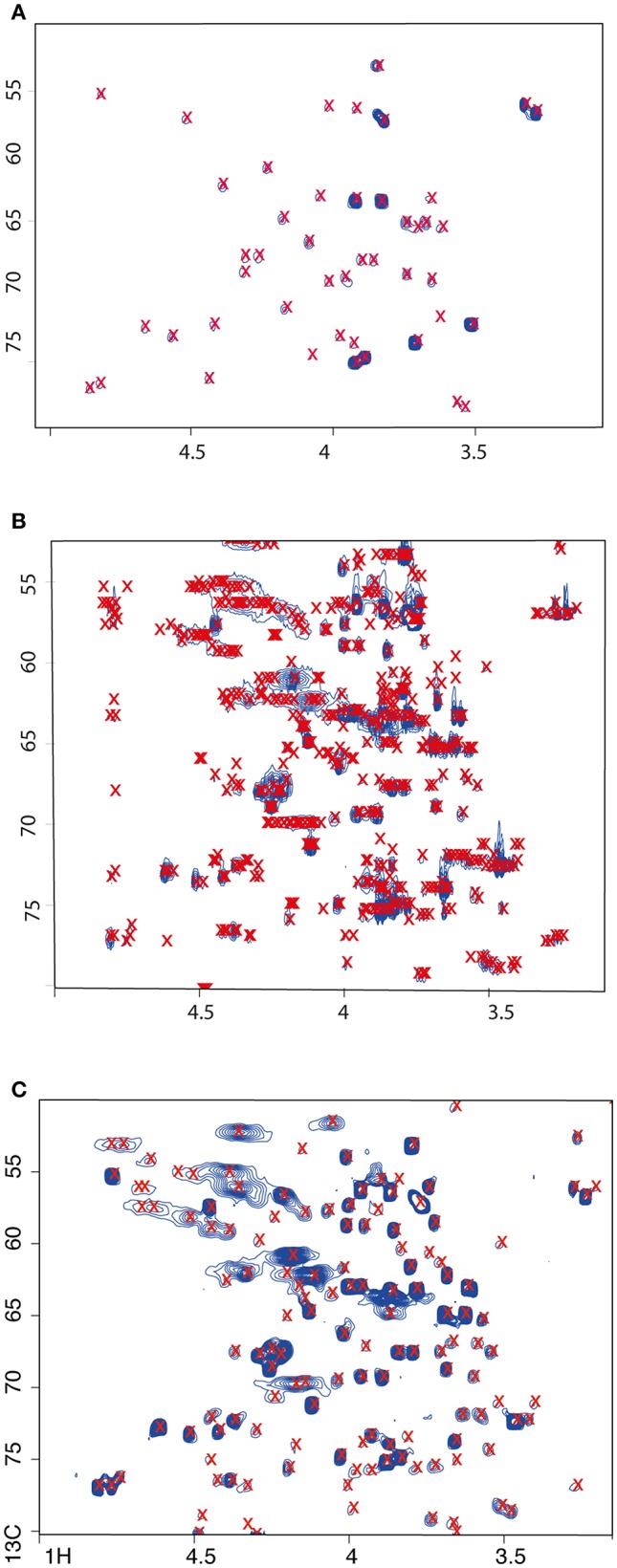
The ct-HSQC **(C)** is better for peak detection using fully ^13^C labeled *C. elegans* extracts than the standard sequence using either labeled **(B)** or unlabeled **(A)** samples. Detected peaks are labeled with a red x. Selected region of spectra shown only.

Here, we carried out a simple automated assignment based on simple matching to reference spectra (taken from the HMDB). The observed advantages of the constant time HSQC over the regular sequence, the simplification of the spectra by collapse of splitting patterns and the increased intensity of the resulting cross peaks, should improve automatic database-assisted compound annotation. We used the package rNMR for peak detection; as expected, the ^12^C HSQC showed the fewest (*n* = 69) detected peaks (Figure 2). In the regular HSQC acquisition, many more peaks (*n* = 1242) were detected than with the constant time version (*n* = 340) (Figure 2). Manual checking of which cross peaks were detected as apparent peaks showed a surprisingly good performance of the algorithm for the ct-HSQC spectrum. Each cross peak was detected once and only once. Even coinciding cross peaks was recognized by the algorithm, which then flagged all apices of a clustered peak group correctly. Conversely, for the regular ^13^C HSQC acquisition, multiple apices were detected for a single cross peak, greatly reducing the value of an automated approach. Close inspection revealed that the algorithm identified the local maxima of the C-C splitting patterns as individual peaks. Tweaking parameters, e.g., by using smoothing filters, did not lead to noticeable improvement. We did not compare multiple software packages for peak detection. Naturally, it is possible that other software packages might have performed better for the standard HSQC, yet it is still likely that the ct-HSQC-like would have worked better simply because automated differentiation between overlapping cross peaks and the maxima of complex splitting patterns is extremely challenging. Given this good performance of the ct-HSQC for peak detection, we only used these data to compare to the unlabelled sample for the matching to actual metabolites.

We aligned the spectra with the reference spectra as much as possible, by removing systematic offsets. We used a constant chemical shift offset of 0.02 ppm in the proton and 0.228 ppm in the carbon domain, compared to the HMDB spectra, as this lowered the root mean square matching error on average between reference and acquired resonances by 3 fold in the proton domain and 0.5 fold in the carbon domain. We then used a Monte-Carlo approach to optimize the “error window” for the spectral matching.

**Figure 3:**
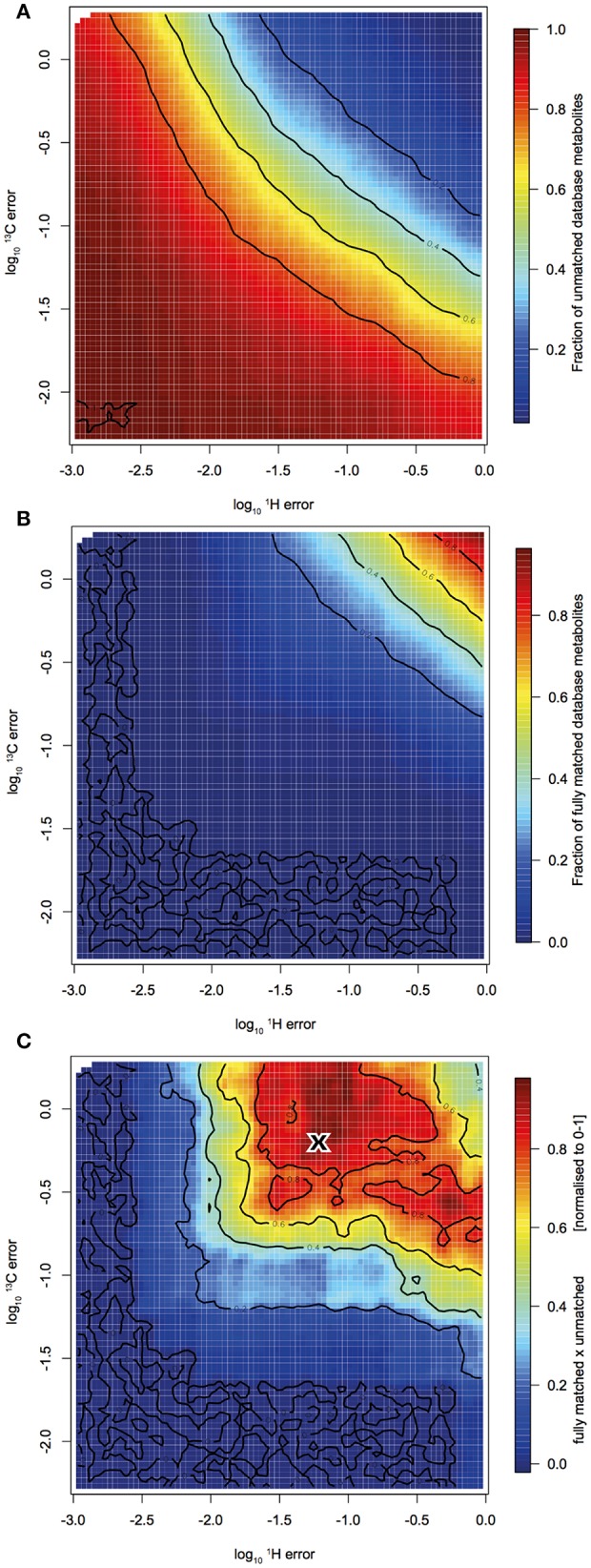
Monte Carlo simulation to find ideal matching error window: HSQC peak - reference spectrum peak matching quality indicators in response to varied proton and carbon matching error. **(A)** Shows the fraction of reference metabolites that were not yet matched with at least one HSQC cross peak. **(B)** Shows the fraction of reference metabolites that were fully matched, i.e., each cross peak of a metabolite is matched to a cross peak in the HSQC spectra. **(C)** is the convolution of **(A,B)**. The “X” denotes the maximum and therefore the optimum matching error window.

Figure 3 displays the empirical relationship between the allowed error in the C and H domain and (a) the fraction of database metabolites yet completely unmatched by any HSQC cross peak and (b) the fraction of fully matched database metabolites, i.e., each cross peak of a reference metabolite is matched to a cross peak from the HSQC spectrum. Interestingly, both functions slopes were aligned diagonally, which means that to achieve the same number of matches, the error tolerance in the proton domain can be increased, if the error tolerance in the carbon domain is increased (and vice versa). In order to determine the best tradeoff between missing metabolites (a) and spurious “over-assignment” (b), we determined the optimum between those two functions by convolution (a * b = c). This gave ± 0.056 ppm and ± 0.56 ppm as matching tolerances in the proton and carbon domains, respectively (Figure 3). These are surprisingly large values compared to error windows that have been used in previous studies.

**Figure 4:**
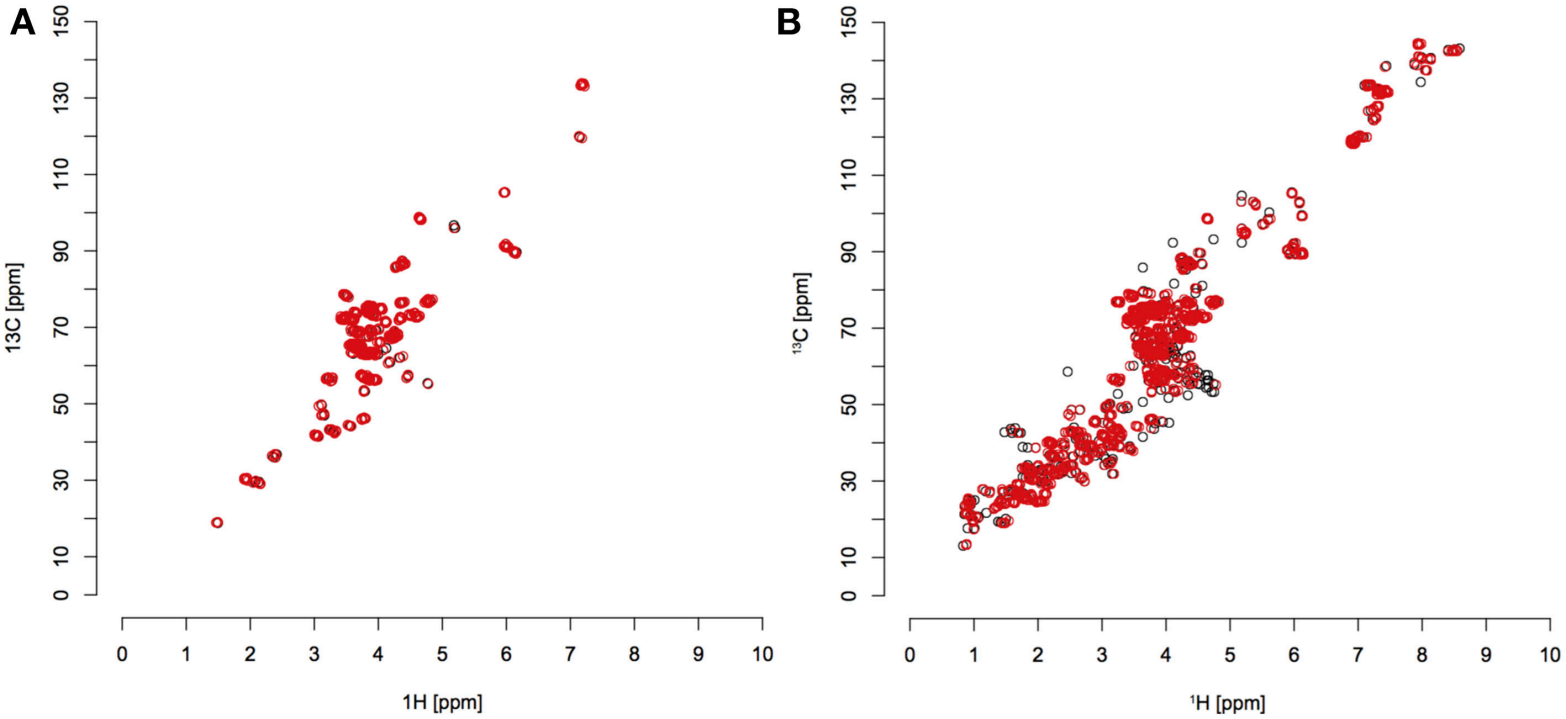
Red circles indicate detected peaks that were matched to reference compounds from the HMDB library; black circles indicate detected peaks that were not matched. **(A)** unlabeled *C. elegans* extract; **(B)** fully ^13^C labeled *C. elegans* extract.

Using these tolerances, a large majority of peaks were matched to references from HMDB. There were still some unmatched peaks, particularly in the ^13^C sample (Figure 4). This could be due to a too small error window, or more likely metabolites that are not recorded in the HMDB. *C. elegans* produces many secondary metabolites, such as ascarosides (Srinivasan et al., 2012).

Using this automated approach, 302 records were matched from the HMDB reference list (Table S1). Not all of these represent plausible matches to metabolites—this is not a fault of the approach, but a simple recognition that any complex matching procedure will be subject to errors. The numbers of false negatives and false positives will decrease and increase, respectively, as less stringent matching criteria are used; exactly where a boundary should be placed depends on the aim of specific experiments. There are 57 metabolites that have 100% of their cross-peaks matched in the ^13^C-enriched sample; however, five of these represent duplicate entries (because D- and L-enantiomers were both included in the reference list), and two are not metabolites (glycogen, pectin). In comparison, the spectrum from the unenriched biomass had only 9 metabolites with 100% of peaks matched. Of the remaining 50 compounds, 38 (including glycogen) are found in the WormJam metabolic model list of metabolites (Witting et al., 2018). The 14 with no match include some which may well be false positives (malonate, dimethylmalonate, tartrate, dimethylamine, dimethylsulfone—although this last metabolite is found in human plasma (Engelke et al., 2005)—particularly as these metabolites have only a single cross-peak, so errors are more likely than for metabolites with multiple peaks). The remaining 8 metabolites include some which are not obviously biologically implausible: glycyl-leucine, N-acetylglutamate, threonate, ribitol, N-acetylaspartate, acetylglycine, acetylphosphate, and glutarate (although the CoA derivative is listed in the model). It is clearly possible that some of these may be real metabolites. Indeed, 6 of the metabolites not included in the model are included in the list of metabolites identified from other metabolomics studies (malonate, dimethylamine, glycylleucine, N-acetylglutamate, N-acetylaspartate, and glutarate), although, as the authors themselves point out, this does not guarantee that the assignments were all reliable (Witting et al., 2018).

Obviously, those metabolites with all peaks matched will tend to be more reliable, but as the list is extended to partial matches, unquestionable false positive hits appear—for instance, cyclohexanone, theophylline, 2,4-dichlorophenol, metoprolol, phenol, sulfamethoxazole-N-glucuronide, and 3-dechloroethylifosfamide. These only appear at lower rank—the highest-ranked of these is phenol, at 111; the others are ranked between 130 to 301. Clearly, all results will need to be manually validated for definitive assignment, including spiking studies and/or independent analytical techniques where appropriate. However, as a rapid and automated first effort at assigning the metabolome of a new matrix, isotope enrichment, acquisition by a ct-HSQC sequence and database matching with optimized parameters, appears to be a valuable workflow. The ct-HSQC may well prove to be a useful complement to other HSQC sequences for metabolite profiling analysis in labeled samples (as opposed to qualitative analysis only), although this remains to be properly evaluated. Recent studies have shown 2D spectra can even be acquired for labeled living animals (Lane et al., 2019), and so having a number of different options for spectral analysis promise to be more useful than ever.

## Author Contributions

FG carried out all experimental work and data analysis. FG and JB drafted the initial version of the manuscript. All authors contributed to planning the experiments and overall project direction, and all authors approved the final submitted manuscript.

### Conflict of Interest Statement

The authors declare that the research was conducted in the absence of any commercial or financial relationships that could be construed as a potential conflict of interest.
