# Supplementary material for: 13C Labeling of Nematode Worms to Improve Metabolome Coverage by Heteronuclear Nuclear Magnetic Resonance Experiments
Source: Front Mol Biosci. 2019 Apr 26;6:27. doi: 10.3389/fmolb.2019.00027 (PMC6498324; doi:10.3389/fmolb.2019.00027)
Supplement: Supplementary file 1 [file Table_1.pdf]

Table S1. List of putative metabolites, identified by matching ct-HSQC peaks with HMDB reference spectra. The metabolites are ordered initially by the percentage of peaks successfully matched, and secondarily by the number of peaks for each metabolite.

No	Metabolite Name	HMDB id	PubChem id	Peaks	% 13C	% 12C
1	Citicoline	HMDB01413	13804	11	100	55
2	D-Glucose	HMDB00122	5793	10	100	30
3	Glycogen	HMDB00757	439177	10	100	25
5	Trehalose	HMDB00975	7427	8	100	100
4	Glycerophosphocholine	HMDB00086	71920	8	100	
6	Cytidine triphosphate	HMDB00082	6176	7	100	43
7	Spermidine	HMDB01257	1102	7	100	29
8	Guanosine diphosphate	HMDB01201	8977	6	100	67
10	Glycerol 3-phosphate	HMDB00126	439162	5	100	100
16	D-Arginine	HMDB03416	71070	5	100	60
12	Ornithine	HMDB00214	6262	5	100	60
9	L-Cystathionine	HMDB00099	439258	5	100	40
15	Spermine	HMDB01256	1103	5	100	40
11	L-Lysine	HMDB00182	5962	5	100	20
13	Glycyl-L-leucine	HMDB00759	92843	5	100	
14	N-Acetylglutamic acid	HMDB01138	185	5	100	
22	D-Xylitol	HMDB02917	6912	4	100	100
18	L-Arginine	HMDB00517	6322	4	100	75
21	Threonic acid	HMDB00943	151152	4	100	75
17	Ribitol	HMDB00508	6912	4	100	50
19	L-2-Hydroxyglutaric acid	HMDB00694	439939	4	100	25
20	L-Valine	HMDB00883	6287	4	100	25
24	L-Glutamic acid	HMDB00148	33032	3	100	100
29	3-Phosphoglyceric acid	HMDB00807	724	3	100	67
23	Glycerol	HMDB00131	753	3	100	67
25	L-Alanine	HMDB00161	5950	3	100	67
27	L-Glutamine	HMDB00641	5961	3	100	67
31	D-Glutamine	HMDB03423	145815	3	100	33
32	D-Leucine	HMDB13773	6106	3	100	
26	L-Asparagine	HMDB00168	6267	3	100	
28	L-Leucine	HMDB00687	6106	3	100	

Continued on next page

Continued from previous page

No	Metabolite Name	HMDB id	PubChem id	Peaks	% 13C	% 12C
30	N-Acetyl-L-aspartic acid	HMDB00812	65065	3	100	
40	Acetylglycine	HMDB00532	10972	2	100	50
44	D-Lactic acid	HMDB01311	61503	2	100	50
37	L-Lactic acid	HMDB00190	107689	2	100	50
36	L-Serine	HMDB00187	5951	2	100	50
45	Putrescine	HMDB01414	1045	2	100	50
46	Acetylphosphate	HMDB01494	186	2	100	
33	Beta-Alanine	HMDB00056	239	2	100	
34	Citric acid	HMDB00094	311	2	100	
43	D-Alanine	HMDB01310	71080	2	100	
35	Ethanolamine	HMDB00149	700	2	100	
41	Glutaric acid	HMDB00661	743	2	100	
42	Hydroxypropionic acid	HMDB00700	68152	2	100	
38	Oxoglutaric acid	HMDB00208	51	2	100	
47	Pectin	HMDB03402	441476	2	100	
39	Phosphoenolpyruvic acid	HMDB00263	1005	2	100	
50	Glycine	HMDB00123	750	1	100	100
52	Succinic acid	HMDB00254	1110	1	100	100
54	Tartaric acid	HMDB00956	444305	1	100	100
48	Acetic acid	HMDB00042	176	1	100	
55	Dihydroxyacetone	HMDB01882	670	1	100	
57	Dimethyl sulfone	HMDB04983	6213	1	100	
49	Dimethylamine	HMDB00087	674	1	100	
56	Dimethylmalonic acid	HMDB02001	11686	1	100	
53	Malonic acid	HMDB00691	867	1	100	
51	Orotic acid	HMDB00226	967	1	100	
58	Uridine diphosphate-N-acetylglucosamine	HMDB00290	445675	15	93	73
59	Maltotetraose	HMDB01296	439639	18	89	39
60	D-Glucuronic acid	HMDB00127	444791	9	89	33
61	D-Maltose	HMDB00163	10991489	17	88	35
63	Adenosine triphosphate	HMDB00538	5957	8	88	75
62	Allocystathionine	HMDB00455	10104953	8	88	25
65	Adenosine 3′,5′-diphosphate	HMDB00061	159296	7	86	71
70	Adenosine phosphosulfate	HMDB01003	10238	7	86	71
71	Guanosine triphosphate	HMDB01273	6830	7	86	71
64	Adenosine monophosphate	HMDB00045	6083	7	86	57
68	Uridine 5′-diphosphate	HMDB00295	6031	7	86	57
66	L-Aspartic acid	HMDB00191	5960	7	86	
69	N-Alpha-acetyllysine	HMDB00446	192590	7	86	

Continued on next page

Continued from previous page

No	Metabolite Name	HMDB id	PubChem id	Peaks	% 13C	% 12C
67	Pantothenic acid	HMDB00210	988	7	86	
72	Alpha-D-Glucose	HMDB03345	79025	13	85	46
75	Guanosine monophosphate	HMDB01397	6804	6	83	50
74	L-Isoleucine	HMDB00172	6306	6	83	
73	L-Phenylalanine	HMDB00159	6140	6	83	
76	Uridine diphosphate glucose	HMDB00286	53477679	17	82	65
81	D-Lysine	HMDB03405	57449	5	80	40
78	Gamma-Glutamylcysteine	HMDB01049	123938	5	80	40
77	Homo-L-arginine	HMDB00670	9085	5	80	20
79	Aminocaproic acid	HMDB01901	564	5	80	
80	Erythrose	HMDB02649	439574	5	80	
82	Maltitol	HMDB02928	493591	14	79	57
83	N1-Acetylspermine	HMDB01186	916	9	78	22
84	Uridine diphosphategalactose	HMDB00302	18068	13	77	54
85	Inosinic acid	HMDB00175	8582	8	75	63
86	Sorbitol	HMDB00247	5780	8	75	63
87	1-Methylguanosine	HMDB01563	96373	8	75	
88	Alpha-Aspartyl-lysine	HMDB04987	6427003	8	75	
92	L-Iditol	HMDB11632	5460044	4	75	75
89	(R)-3-Hydroxyisobutyric acid	HMDB00336	11217234	4	75	
91	3-Aminoisobutanoic acid	HMDB03911	64956	4	75	
90	Citrulline	HMDB00904	9750	4	75	
94	Beta-N-Acetylglucosamine	HMDB00803	24139	15	73	60
93	N-Acetyl-D-glucosamine	HMDB00215	11861101	15	73	60
95	Maltotriose	HMDB01262	439586	18	72	39
97	Glucose 1-phosphate	HMDB01586	439165	7	71	43
96	Isovalerylglutamic acid	HMDB00726	133383	7	71	
98	NAD	HMDB00902	5893	19	68	32
99	D-Xylose	HMDB00098	135191	18	67	22
100	7-Methylguanosine	HMDB01107	445404	9	67	22
102	Glutathione	HMDB00125	124886	6	67	50
106	Gluconic acid	HMDB00625	10690	6	67	17
104	Gluconolactone	HMDB00150	7027	6	67	17
105	N6-Acetyl-L-lysine	HMDB00206	92832	6	67	17
103	Guanosine	HMDB00133	6802	6	67	
101	Iodotyrosine	HMDB00021	439744	6	67	
107	N-Acetyl-L-tyrosine	HMDB00866	68310	6	67	
121	Phosphorylcholine	HMDB01565	1014	3	67	100
128	D-Threitol	HMDB04136	169019	3	67	67

Continued on next page

Continued from previous page

No	Metabolite Name	HMDB id	PubChem id	Peaks	% 13C	% 12C
108	L-Carnitine	HMDB00062	2724480	3	67	67
110	L-Threonine	HMDB00167	6288	3	67	67
125	Canavanine	HMDB02706	439202	3	67	33
119	Diaminopimelic acid	HMDB01370	439283	3	67	33
117	Homocysteine	HMDB00742	778	3	67	33
118	Propionylglycine	HMDB00783	98681	3	67	33
131	3-Dechloroethylifosfamide	HMDB13858	1983	3	67	
120	3-Hydroxyanthranilic acid	HMDB01476	86	3	67	
127	4-Guanidinobutanoic acid	HMDB03464	500	3	67	
129	4-Heptanone	HMDB04814	31246	3	67	
115	4-Hydroxyphenylpyruvic acid	HMDB00707	979	3	67	
123	Acetaminophen	HMDB01859	1983	3	67	
124	Acetylcysteine	HMDB01890	12035	3	67	
113	D-Alpha-aminobutyric acid	HMDB00650	439691	3	67	
122	DL-O-Phosphoserine	HMDB01721	106	3	67	
109	Gamma-Aminobutyric acid	HMDB00112	119	3	67	
114	Ketoleucine	HMDB00695	70	3	67	
112	L-Alpha-aminobutyric acid	HMDB00452	80283	3	67	
126	L-Dihydroorotic acid	HMDB03349	439216	3	67	
116	Levulinic acid	HMDB00720	11579	3	67	
111	Phenol	HMDB00228	996	3	67	
130	Sulfamethoxazole N1-glucuronide	HMDB13855	996	3	67	
134	Glucose 6-phosphate	HMDB01401	5958	11	64	36
133	N-Acetylmannosamine	HMDB01129	11096158	11	64	36
132	NADP	HMDB00217	5886	11	64	27
139	Uridine 5′-monophosphate	HMDB00288	6030	8	63	63
137	D-Mannose	HMDB00169	18950	8	63	50
135	Argininosuccinic acid	HMDB00052	16950	8	63	38
138	Saccharopine	HMDB00279	160556	8	63	38
136	Cytidine monophosphate	HMDB00095	6131	8	63	25
140	Cysteineglutathione disulfide	HMDB00656	53477713	10	60	30
146	Hydroxyphenyllactic acid	HMDB00755	9378	5	60	40
149	L-Arabitol	HMDB01851	439255	5	60	40
143	2-Hydroxyadipic acid	HMDB00321	193530	5	60	20
151	3-Phenoxypropionic acid	HMDB02229	81596	5	60	20
148	6-Phosphogluconic acid	HMDB01316	91493	5	60	20
145	Homocitrulline	HMDB00679	65072	5	60	20
142	L-Tyrosine	HMDB00158	6057	5	60	20
150	N-Acetylputrescine	HMDB02064	122356	5	60	20

Continued on next page

Continued from previous page

No	Metabolite Name	HMDB id	PubChem id	Peaks	% 13C	% 12C
141	(S)-3-Hydroxyisobutyric acid	HMDB00023	440873	5	60	
144	Leucinic acid	HMDB00665	92779	5	60	
147	Ureidosuccinic acid	HMDB00828	93072	5	60	
152	N-Acetylgalactosamine 4-sulphate	HMDB00781	446101	12	58	33
153	Allose	HMDB01151	12285879	7	57	29
154	5-Phenylvaleric acid	HMDB02043	16757	7	57	
155	Coenzyme A	HMDB01423	6816	16	56	25
156	Inosine triphosphate	HMDB00189	8583	9	56	56
159	Mannose 6-phosphate	HMDB01078	439198	9	56	33
157	Aminoadipic acid	HMDB00510	469	9	56	11
160	D-Pantethine	HMDB03828	452306	9	56	
158	L-Aspartyl-L-phenylalanine	HMDB00706	93078	9	56	
161	Cellobiose	HMDB00055	10712	20	55	45
162	Alpha-Lactose	HMDB00186	84571	20	55	30
164	Furan	HMDB13785	5988	11	55	27
163	Sucrose	HMDB00258	5988	11	55	27
165	L-Arabinose	HMDB00646	439195	12	50	25
166	Homocarnosine	HMDB00745	10243361	8	50	13
167	5-Methoxydimethyltryptamine	HMDB02004	1832	8	50	
177	D-Xylulose	HMDB01644	5289590	6	50	50
170	L-Acetylcarnitine	HMDB00201	1	6	50	17
171	2-Methyl-3-ketovaleric acid	HMDB00408	189028	6	50	
168	3-Methoxytyramine	HMDB00022	1669	6	50	
173	3-Methyl-2-oxovaleric acid	HMDB00491	47	6	50	
172	3,4-Dihydroxyhydrocinnamic acid	HMDB00423	348154	6	50	
174	DL-Dopa	HMDB00609	836	6	50	
169	L-Dopa	HMDB00181	6047	6	50	
176	N-Formyl-L-methionine	HMDB01015	6995182	6	50	
175	Valerylglycine	HMDB00927	4737557	6	50	
195	2,3-Butanediol	HMDB03156	262	4	50	25
181	Glutarylglycine	HMDB00590	23592950	4	50	25
183	Isovalerylglycine	HMDB00678	546304	4	50	25
185	L-Homoserine	HMDB00719	12647	4	50	25
184	L-Methionine	HMDB00696	6137	4	50	25
192	Methionine sulfoxide	HMDB02005	847	4	50	25
193	N-Methyl-D-aspartic acid	HMDB02393	22880	4	50	25
187	Neopterin	HMDB00845	4455	4	50	25
186	Pyrrolidonecarboxylic acid	HMDB00805	499	4	50	25
179	Tyramine	HMDB00306	5610	4	50	25

Continued on next page

Continued from previous page

No	Metabolite Name	HMDB id	PubChem id	Peaks	% 13C	% 12C
190	2-Ketohexanoic acid	HMDB01864	159664	4	50	
180	4,5-Dihydroorotic acid	HMDB00528	648	4	50	
196	5-Aminopentanoic acid	HMDB03355	138	4	50	
191	D-threo-Isocitric acid	HMDB01874	5318532	4	50	
188	Histamine	HMDB00870	774	4	50	
194	Methylimidazoleacetic acid	HMDB02820	75810	4	50	
178	Phenylacetic acid	HMDB00209	999	4	50	
182	Succinylacetone	HMDB00635	5312	4	50	
189	trans-Cinnamic acid	HMDB00930	444539	4	50	
200	Betaine	HMDB00043	247	2	50	100
202	Creatine	HMDB00064	586	2	50	50
219	N-Methyl-a-aminoisobutyric acid	HMDB02141	6951124	2	50	50
214	Phosphocreatine	HMDB01511	587	2	50	50
197	1,3-Diaminopropane	HMDB00002	428	2	50	
198	2-Ketobutyric acid	HMDB00005	58	2	50	
217	2,2-Dimethylsuccinic acid	HMDB02074	11701	2	50	
209	5-Hydroxymethyluracil	HMDB00469	78168	2	50	
220	A-Ketoglutaric acid oxime	HMDB02467	13012726	2	50	
201	Acetoacetic acid	HMDB00060	96	2	50	
215	Acetone	HMDB01659	180	2	50	
208	Adipic acid	HMDB00448	196	2	50	
222	Diethanolamine	HMDB04437	8113	2	50	
203	Dihydrouracil	HMDB00076	649	2	50	
204	Dimethylglycine	HMDB00092	673	2	50	
211	Galactaric acid	HMDB00639	3037582	2	50	
216	Isobutyric acid	HMDB01873	6590	2	50	
212	Isopropyl alcohol	HMDB00863	3776	2	50	
210	L-Cysteine	HMDB00574	5862	2	50	
221	Phosphonoacetate	HMDB04110	546	2	50	
205	Propionic acid	HMDB00237	1032	2	50	
213	Pyrocatechol	HMDB00957	289	2	50	
207	Sarcosine	HMDB00271	1088	2	50	
218	Syringic acid	HMDB02085	10742	2	50	
206	Taurine	HMDB00251	1123	2	50	
199	Ureidopropionic acid	HMDB00026	111	2	50	
223	5-Hydroxylysine	HMDB00450	3032849	9	44	33
224	dATP	HMDB01532	15993	9	44	22
232	D-Tagatose	HMDB03418	92092	7	43	43
233	5-Methoxytryptamine	HMDB04095	1833	7	43	14

Continued on next page

Continued from previous page

No	Metabolite Name	HMDB id	PubChem id	Peaks	% 13C	% 12C
225	Carnosine	HMDB00033	439224	7	43	14
227	Glucosan	HMDB00640	2724705	7	43	14
228	Suberylglycine	HMDB00953	6453952	7	43	14
229	2′-Deoxyguanosine 5′-monophosphate	HMDB01044	65059	7	43	
230	dGTP	HMDB01440	65103	7	43	
231	Diphenhydramine	HMDB01927	3100	7	43	
234	o-Tyrosine	HMDB06050	91482	7	43	
226	Xanthosine	HMDB00299	64959	7	43	
236	Glucosamine	HMDB01514	45933887	12	42	25
235	2-Methylbutyroylcarnitine	HMDB00378	6426901	12	42	17
237	Lactulose	HMDB00740	11024312	22	41	14
239	3,4-Dihydroxyphenylglycol	HMDB00318	91528	5	40	20
241	D-Arabitol	HMDB00568	827	5	40	20
247	L-Norleucine	HMDB01645	21236	5	40	20
242	Ortho-Hydroxyphenylacetic acid	HMDB00669	11970	5	40	20
245	Phenylacetylglycine	HMDB00821	68144	5	40	20
240	2-Hydroxy-3-methylbutyric acid	HMDB00407	99823	5	40	
238	Dopamine	HMDB00073	681	5	40	
244	Hydrocinnamic acid	HMDB00764	107	5	40	
243	Hydroxyisocaproic acid	HMDB00746	83697	5	40	
246	trans-Ferulic acid	HMDB00954	445858	5	40	
248	Stachyose	HMDB03553	439531	23	39	22
249	NADH	HMDB01487	928	18	39	28
253	Ribothymidine	HMDB00884	445408	8	38	38
251	Uridine	HMDB00296	6029	8	38	38
255	5-Methylcytidine	HMDB00982	92918	8	38	25
250	Cytidine	HMDB00089	6175	8	38	25
256	dCMP	HMDB01202	13945	8	38	13
252	Isobutyryl-L-carnitine	HMDB00736	10177002	8	38	
254	L-Tryptophan	HMDB00929	6305	8	38	
257	D-Galactose	HMDB00143	439357	11	36	9
258	L(-)-Nicotine pestanal	HMDB01934	89594	11	36	
259	1-Methyladenosine	HMDB03331	27476	17	35	12
260	Pteroyltriglutamic acid	HMDB01902	6968	15	33	
261	Metoprolol	HMDB01932	4171	12	33	8
262	Fructose 6-phosphate	HMDB00124	69507	9	33	22
263	Inosine	HMDB00195	6021	9	33	11
267	3-Methylhistidine	HMDB00479	64969	6	33	17
265	L-Histidine	HMDB00177	6274	6	33	17

Continued on next page

Continued from previous page

No	Metabolite Name	HMDB id	PubChem id	Peaks	% 13C	% 12C
266	Serotonin	HMDB00259	5202	6	33	17
271	3-Chlorotyrosine	HMDB01885	119226	6	33	
272	3-Phenylbutyric acid	HMDB01955	20724	6	33	
264	Epinephrine	HMDB00068	5816	6	33	
269	L-3-Phenyllactic acid	HMDB00748	643327	6	33	
268	L-Kynurenine	HMDB00684	161166	6	33	
270	Phenyllactic acid	HMDB00779	3848	6	33	
288	2,3-Diaminopropionic acid	HMDB02006	97328	3	33	67
278	6-Dimethylaminopurine	HMDB00473	3134	3	33	33
274	Choline	HMDB00097	305	3	33	33
294	Erythritol	HMDB02994	222285	3	33	33
275	Galactitol	HMDB00107	11850	3	33	33
273	p-Hydroxyphenylacetic acid	HMDB00020	127	3	33	33
292	1,11-Undecanedicarboxylic acid	HMDB02327	10458	3	33	
289	2-Pyrrolidinone	HMDB02039	12025	3	33	
293	2,4-Diaminobutyric acid	HMDB02362	470	3	33	
301	2,4-Dichlorophenol	HMDB04811	8449	3	33	
286	5-Aminolevulinic acid	HMDB01149	137	3	33	
296	Acetoin	HMDB03243	179	3	33	
291	Cadaverine	HMDB02322	273	3	33	
302	cis-4-Octenedioic acid	HMDB04982	11805205	3	33	
297	Cyclohexanone	HMDB03315	7967	3	33	
281	Cysteine-S-sulfate	HMDB00731	115015	3	33	
298	D-Cysteine	HMDB03417	92851	3	33	
279	Gamma-Butyrolactone	HMDB00549	7302	3	33	
295	Guanidinosuccinic acid	HMDB03157	439918	3	33	
280	Isovaleric acid	HMDB00718	10430	3	33	
290	Itaconic acid	HMDB02092	811	3	33	
299	L-Allothreonine	HMDB04041	99289	3	33	
277	L-Cystine	HMDB00192	67678	3	33	
276	L-Malic acid	HMDB00156	222656	3	33	
282	Malic acid	HMDB00744	525	3	33	
283	Pimelic acid	HMDB00857	385	3	33	
300	Selenocystine	HMDB04122	15104	3	33	
285	Suberic acid	HMDB00893	10457	3	33	
287	Theophylline	HMDB01889	2153	3	33	
284	Undecanedioic acid	HMDB00888	15816	3	33	
