# Supplementary material for: 13C Labeling of Nematode Worms to Improve Metabolome Coverage by Heteronuclear Nuclear Magnetic Resonance Experiments
Source: Front Mol Biosci. 2019 Apr 26;6:27. doi: 10.3389/fmolb.2019.00027 (PMC6498324; doi:10.3389/fmolb.2019.00027)

Figure S1. A: HSQC of unlabelled C. elegans extract. B: ct-HSQC of 13C-labelled extract. C: HSQC of labelled extract using standard sequence.

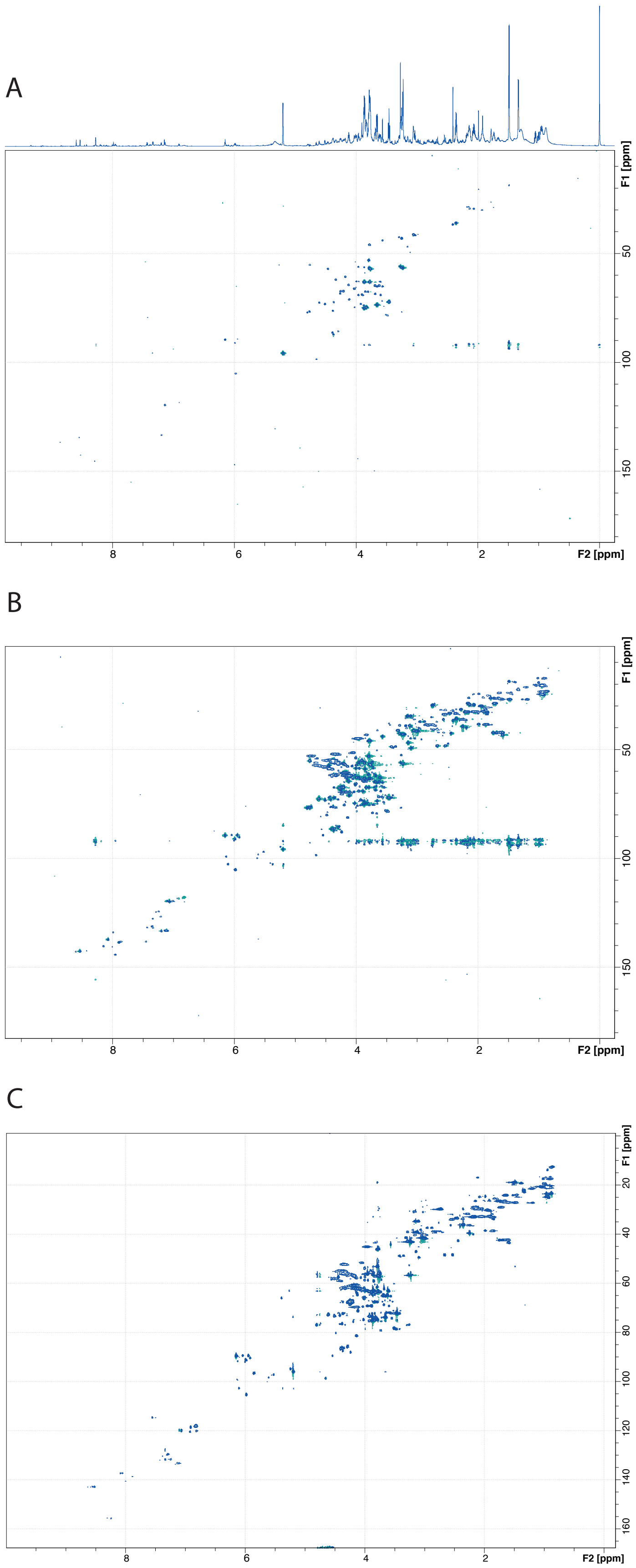

Supplement: Supplementary file 2 [file Image_1.pdf]
