# Supplementary figures and images for: 13C Labeling of Nematode Worms to Improve Metabolome Coverage by Heteronuclear Nuclear Magnetic Resonance Experiments
Source: Front Mol Biosci. 2019 Apr 26;6:27. doi: 10.3389/fmolb.2019.00027 (PMC6498324; doi:10.3389/fmolb.2019.00027)

Figure S2. Expanded region of regular (A) and ct-HSQC (B) of fully-labelled *C. elegans* extracts.

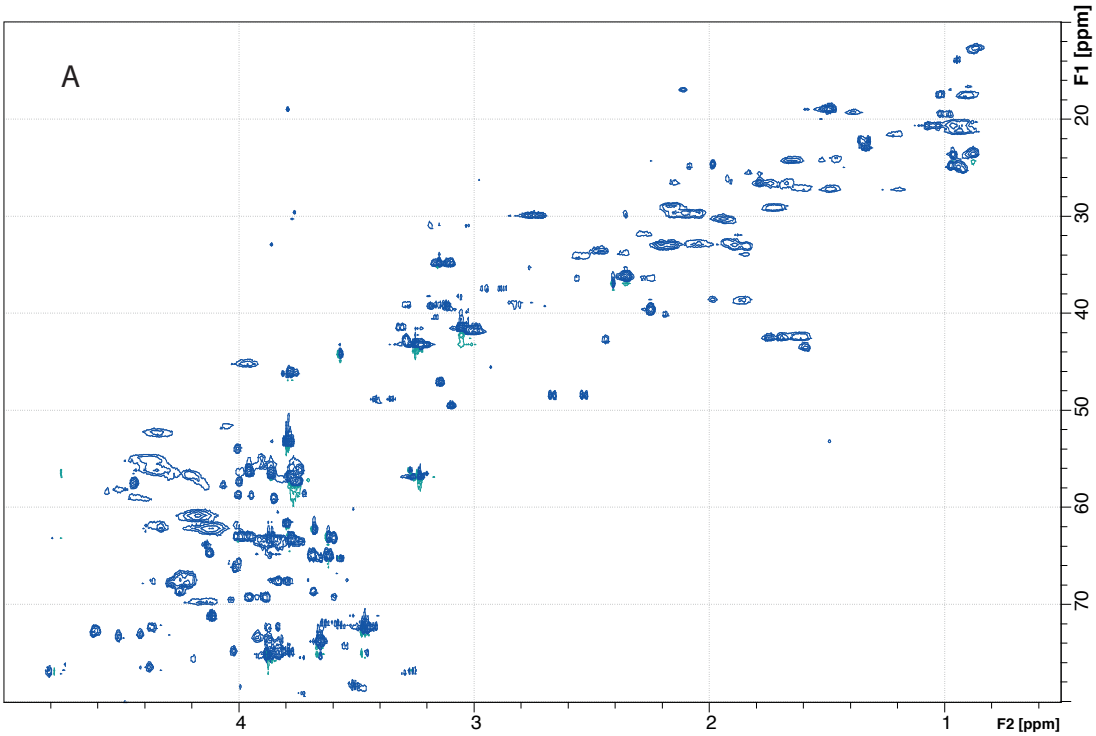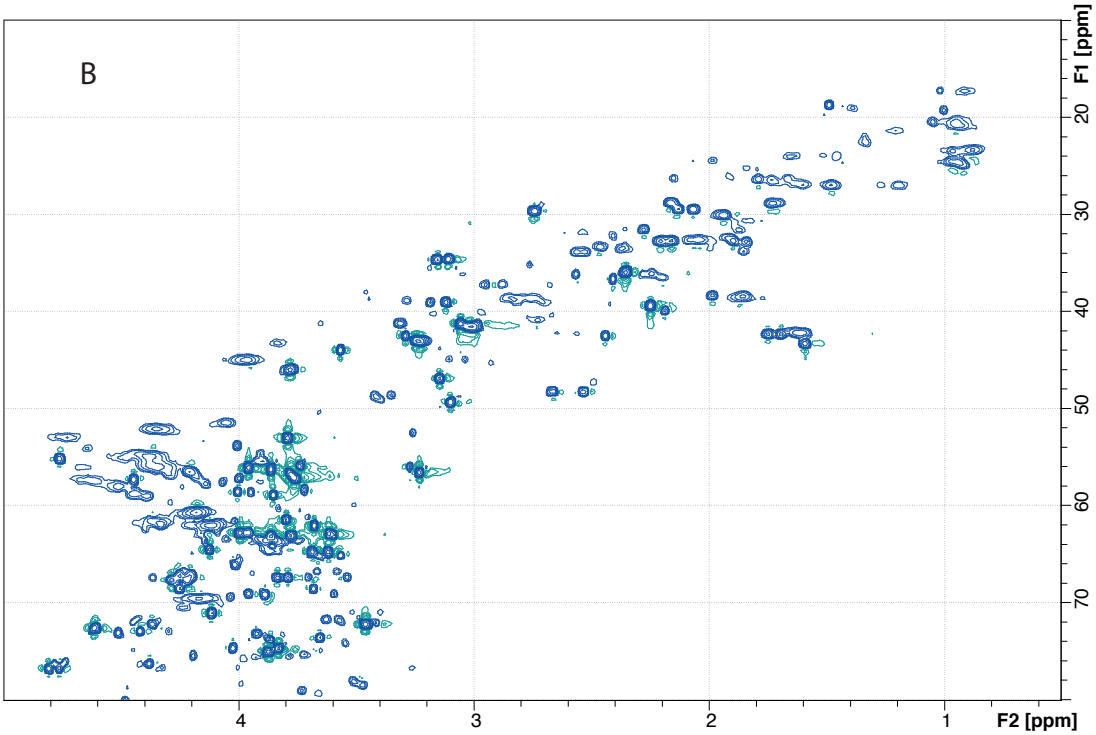

Supplement: Supplementary file 3 [file Image_2.pdf]
