# Supplementary material for: 13C Labeling of Nematode Worms to Improve Metabolome Coverage by Heteronuclear Nuclear Magnetic Resonance Experiments
Source: Front Mol Biosci. 2019 Apr 26;6:27. doi: 10.3389/fmolb.2019.00027 (PMC6498324; doi:10.3389/fmolb.2019.00027)

Figure S3. The ct-HSQC (a) has reduced sensitivity compared to the standard HSQC experiment (b): as illustrated here, the peak at 6.06/88.9 ppm is not visible in the ct-HSQC spectrum.

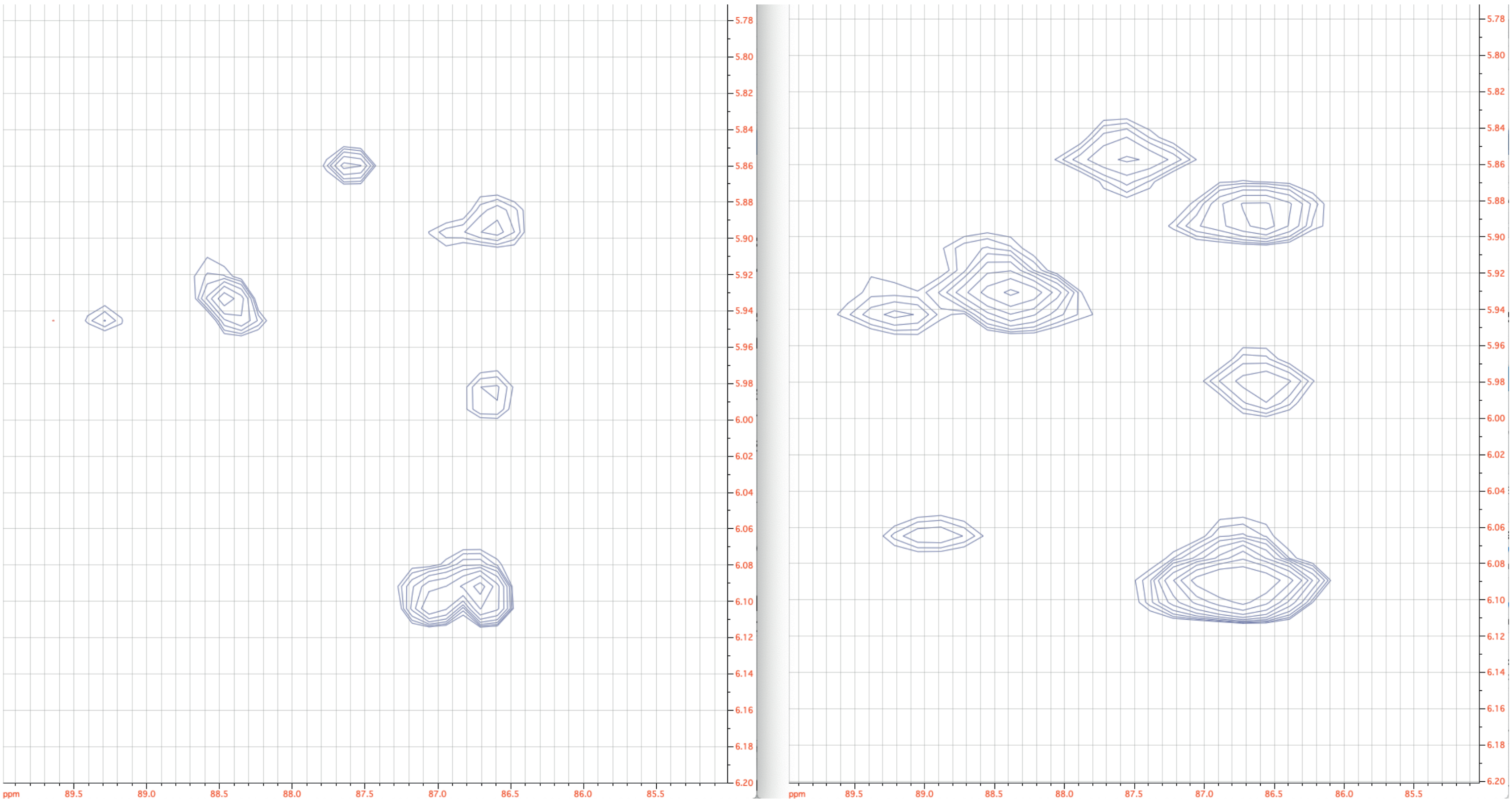

Supplement: Supplementary file 4 [file Image_3.pdf]
